# Racial disparities in adult all-cause and cause-specific mortality among us adults: mediating and moderating factors

**DOI:** 10.1186/s12889-016-3744-z

**Published:** 2016-10-22

**Authors:** M. A. Beydoun, H. A. Beydoun, N. Mode, G. A. Dore, J. A. Canas, S. M. Eid, A. B. Zonderman

**Affiliations:** 1 NIH Biomedical Research Center, National Institute on Aging, IRP, 251 Bayview Blvd. Suite 100 Room #:04B118, Baltimore, MD 21224 USA; 2 Department of Medicine, Johns Hopkins University School of Medicine, Baltimore, MD USA; 3 Pediatric Endocrinology, Diabetes and Metabolism Nemours Children’s Clinic, Jacksonville, FL USA

**Keywords:** Race/ethnicity, Socio-economic status, Adult mortality, Cancer, Cardiovascular disease

## Abstract

**Background:**

Studies uncovering factors beyond socio-economic status (SES) that would explain racial and ethnic disparities in mortality are scarce.

**Methods:**

Using prospective cohort data from the Third National Health and Nutrition Examination Survey (NHANES III), we examined all-cause and cause-specific mortality disparities by race, mediation through key factors and moderation by age (20–49 vs. 50+), sex and poverty status. Cox proportional hazards, discrete-time hazards and competing risk regression models were conducted (*N* = 16,573 participants, *n* = 4207 deaths, Median time = 170 months (1–217 months)).

**Results:**

Age, sex and poverty income ratio-adjusted hazard rates were higher among Non-Hispanic Blacks (NHBs) vs. Non-Hispanic Whites (NHW). Within the above-poverty young men stratum where this association was the strongest, the socio-demographic-adjusted HR = 2.59, *p* < 0.001 was only partially attenuated by SES and other factors (full model HR = 2.08, *p* = 0.003). Income, education, diet quality, allostatic load and self-rated health, were among key mediators explaining NHB *vs*. NHW disparity in mortality. The Hispanic paradox was observed consistently among women above poverty (young and old). NHBs had higher CVD-related mortality risk compared to NHW which was explained by factors beyond SES. Those factors did not explain excess risk among NHB for neoplasm-related death (fully adjusted HR = 1.41, 95 % CI: 1.02–2.75, *p* = 0.044). Moreover, those factors explained the lower risk of neoplasm-related death among MA compared to NHW, while CVD-related mortality risk became lower among MA compared to NHW upon multivariate adjustment.

**Conclusions:**

In sum, racial/ethnic disparities in all-cause and cause-specific mortality (particularly cardiovascular and neoplasms) were partly explained by socio-demographic, SES, health-related and dietary factors, and differentially by age, sex and poverty strata.

**Electronic supplementary material:**

The online version of this article (doi:10.1186/s12889-016-3744-z) contains supplementary material, which is available to authorized users.

## Background

The past several decades have witnessed an overall reduction in mortality rates coupled with a sustained Black-White mortality rate disparity with higher rates observed in Blacks (e.g. 1950: 5310 per 100,000 resident Non-Hispanic Black (NHB) population over 65y vs. 4865 per 100,000 resident Non-Hispanic White (NHW) population over 65y; 2006: Rates were 3669 per 100,000 vs. 2456 per 100,000 in NHB vs. NHW, respectively) [1, 2]. Though narrowing down in recent years, [3] this disparity in mortality remains wide and was explained partly by a long-standing socioeconomic racial stratification [4]. This comes in stark contrast to the Mexican-American (MA) and Hispanic ethnic group disparities with NHWs in mortality rates. In fact, assuming socioeconomic resources are equalized, mortality rate among MAs was consistently lower compared to NHWs a phenomenon known as the Hispanic paradox [3, 5–10]. Thus, in the absence of socio-economic equality, sizeable Black-White disparities in mortality will persist, while MAs would live longer than NHWs under similar socio-economic conditions [3, 9]. While socio-economic status (SES) was shown to moderate as well as mediate racial disparities in adult mortality, [11] only one study to date has systematically uncovered the contribution of behavioral and health-related potential mediators that go beyond SES [12].

We used national data to evaluate race/ethnicity effects on all-cause and cause-specific adult mortality, while stratifying by age group, sex and poverty status for all-cause mortality. We further evaluated the putative mediating effects of SES, lifestyle, social support and health-related factors in the association between race and all-cause mortality risk and examined cause-of-death structure across racial and ethnic groups.

## Methods

### Study population

The National Center for Health Statistics (NCHS) of the Centers for Disease Control and Prevention (CDC) conducted the NHANES III (Phase I: 1988–1991, Phase II: 1991–1994) by applying a complex multistage probability sample design, providing national estimates of health and nutritional status of the civilian non-institutionalized population [13]. Using questionnaire, physical examination and laboratory data from NHANES III, we retained adults age ≥ 20y (*n* = 18,825), who self-reported their race/ethnicity as NHW, NHB or MA, thus excluding other ethnicities. This yielded a final sample of 18,110 participants, of whom *N* = 16,573 were retained upon multiple imputations for descriptive statistics and *N* = 15,889 for survival analyses, with a total of 4,207 deaths (2359 among NHW, 1090 among NHB and 758 among MA). NHANES III is compliant with the ethical rules for human experimentation stated in the Declaration of Helsinki, including approval of an institutional review board and informed written consent. The current study was approved by the Office of Human Subjects Research Protections, National Institutes of Health.

### Mortality outcome

The NCHS conducted a mortality linkage for NHANES III with the National Death Index (NDI), allowing investigation of baseline characteristics (1988–1994) in relation to mortality rates at follow-up through December 31, 2006. This public-use linked mortality file included eligibility status, assigned vital status, mortality source, person-months of follow-up from interview date and from Mobile Examination Center (MEC)/home exam date, and the underlying or multiple causes of death [14]. We defined an event as death from any cause, starting from the MEC examination date and ending on or before December 31, 2006; or death from the following underlying causes: “Major cardiovascular disease” (International Classification of Diseases, 10th version, (ICD-10) codes: I00-I78), “Neoplasms” (ICD-10 codes: C00-C97, D50-D64), “Diabetes” as an underlying/contributing cause (ICD-10 codes: E10-E14), “Other causes” (all other ICD-10 codes) [14]. “Other causes” were sub-divided into: “infections and respiratory (A..-B.. and J.. codes), “gastrointestinal (GI), Kidney and urological” (K.. and N.. codes), “Neurological disease” (G.. codes), unintentional/intentional injuries (V..-Y..) and “others” (all other codes). This detailed subdivision of causes (8 groups) was used only in part of the analysis whereby the cause of death structure (proportionate mortality) was compared between the three major race/ethnicity groups. In the main analysis of survival data, only deaths from major cardiovascular disease and neoplasms were examined.

### Main predictors

In the selected sample, race/ethnicity was coded as NHW, NHB and MA. Two dummy variables were created to contrast NHB and MA with NHW.

### Exogenous variables

In the final models, exogenous variables were the variables that were allowed to predict both potential mediators and the final outcome. Those included continuous age (y), sex, marital status (1 = Never married, 2 = Married, 3 = Divorced, 4 = Widowed, 5 = Other), household size and urban-rural residence (1 = Urban, 2 = Rural).

### Potential mediators

#### Socio-economic status

Socio-economic status was measured alternatively by continuous poverty income ratio, education (years) and health insurance status (1 = yes, 0 = no).

#### Lifestyle and social support factors

Several factors were included in our models as mediators that could be directly affected by socio-economic status. Those include the latent constructs of “substance abuse”, “nutritional factors”, “physical activity”, “smoking” and “social support”. Substance abuse was operationalized as “alcohol consumption (g/d)” and drug use (1 = Ever, 0 = never); “nutritional factors” as 1995-Healthy Eating Index (1995-HEI) ranging from 0-100, [15] and the mean adequacy ratio score (MAR), [16–18] (Additional file 1); “Physical activity” as 3 related items: Item 1: “Compare activity for past month to past yr (0 = less, 1 = same, 2 = more), item 2: “Active compared with men/women your age” (0 = less, 1 = same, 2 = more), item 3: “Active now compared with self, 10 year ago” (0 = less, 1 = same, 2 = more); “Smoking” with two items: Item 1: “number of cigarettes smoked per day” (0 among non-smokers); item 2: “years smoked cigarettes” (0 among non-smokers); social support with 5 items namely, (1) “In a typical week, how many times do you talk on the telephone with family, friends, or neighbors?”, (2) “How often do you get together with friends or relatives; I mean things like going out together or visiting in each other's homes? (per year)”, (3) “About how often do you visit with any of your other neighbors, either in their homes or in your own? (per year)”, (4) “How often do you attend church or religious services? (per year)”, (5)“Altogether, how often do you attend meetings of the clubs or organizations (per year)”.

#### Health-related factors

The construct of “Health” was operationalized with self-rated health, co-morbidity index and the allostatic load (AL) score. Participants self-rated their health as: “Excellent” (referent), “Very good”, “Good”, “Fair” or “Poor”, while the co-morbidity index consisted of total sum score of 14 possible self-reported conditions, namely “arthritis“, “congestive heart failure“, “stroke“, “asthma“, “chronic bronchitis“, “emphysema“, “hay fever“, “cataracts“, “goiter“, “thyroid disease“, “lupus“, “gout“, “skin cancer”, “other cancer”. The allostatic load total score (0-9) is composed of 9 items which are described in details in Additional file 1 [19].

### Statistical analysis

Using Stata 14.0 (StataCorp, College Station, TX), [20] analyses accounted for survey design complexity [14], by incorporating sampling weights, primary sampling units and strata. Multivariate imputed data [21, 22] was used to estimate means and proportions across race/ethnic groups, as well as various measures of associations including odds ratios and linear regression coefficients, after adjusting for sampling design complexity with survey (svy) commands. Comparison across race/ethnicity groups were made using svy:reg and svy:mlogit commands with race/ethnicity dummy variables as the only predictors. Standard errors were estimated using Taylor series linearization [14]. Comparison between race groups (NHB vs. NHW and MA vs. NHW) in terms of cause-of-death structure was done among all deaths in the selected sample, comparing proportions of each cause by use of a logistic regression model that took into account design complexity and multiple imputations.

Defining time-to-event from any age ≥ 20y since baseline visit (i.e. delayed entry) until death or censoring, we conducted Cox proportional hazards models for all-cause mortality stratifying by age group, sex and poverty status as well as competing risk regression models for cause-specific mortality. The time of follow-up is expressed in months. A series of nested models accounting for sampling weights were carried out and using the imputed data, in which socio-demographic, SES, lifestyle, social support and health-related factors were entered consecutively. The mediating effect of each of those factors was tested more thoroughly in a separate analysis using discrete-time survival analysis within a structural equations modeling (SEM) framework that accounted for sampling weights, though using the original un-imputed data. This was suggested as the optimal method to examine causal mediation within the context of survival analysis [12]. In this generalized SEM model, the final outcome was the hazard rate of death from all-causes and the data structure is person-period rather than participant-level data. The mortality outcome Y (0 = alive, 1 = dead of any cause or specific cause) was modeled using discrete-time survival with a logit link, by adding 18 dummy variables for year of follow-up as the main predictors for risk of death in the person-period modified data. Race/ethnicity was the primary exposure with two contrasts (NH black vs. NH whites; MA vs. NH white). A series of generalized SEM models were conducted, in which alternative mediators “M” were included, one at a time. Those mediators could be grouped under SES, substance abuse, diet, PA, smoking, social support and health. The generalized SEM models included covariates (age, sex, marital status, household size and urban/rural area of residence) that were exogenous to the system along with the race contrast variable. Direct and indirect effects of race were estimated from which the mediation proportion, a non-linear combination of the two, was also estimated using the delta method. Of particular interest were MP > 10 %, indicating an appreciable proportion of a total effect mediated by “M” [23]. Type I error was set at 0.05 in all other analyses.

## Result

### Study sample characteristics by race/ethnicity group

Weighted proportions of NHW, NHB and MA were ~83 %, 12 % and 5 %, respectively. Compared to NHW, NHB and MA participants were younger on average (42y and 38y vs. 46y). NHB had lower proportion male than NHW with the reverse being true for MA. Mean PIR was lower among NHB and MA vs. NHW (2.02 and 1.77 vs. 3.28, *p* < 0.001); with a similar pattern observed for poverty status and education(y). NHB and MA had a higher likelihood of urban residence and a larger mean household size compared to NHW, with marked racial/ethnic difference in marital status. Lack of health insurance was more common in MA (21.1 %) and NHB (9.3 %) vs. NHW (6.1 %), with both NHBs and MA being less likely to rate their health as “Excellent”. Conversely, both mean daily use and years of cigarette used were higher in NHWs, who were nonetheless more physically active than NHBs and MA, based on an item comparing activity to age peers. Drug ever use was highest among NHB (40.2 %), followed by NHW (37.0 %) and the lowest prevalence was among MA (29.4 %). While alcohol consumption did not differ between race groups, both 1995-HEI and MAR indicated poorer overall dietary quality among NHB compared to NHW. With only one exception (clubs and organizations), NHB had more social support from family, friends, neighbors and church, compared to NHW; with the reverse being true for MA. Self-reported co-morbidity was highest among NHW and lowest in MA. In contrast, total AL score based on objective measures of metabolic and inflammatory disturbance markers was higher among NHB than NHW, with no disparity detected between MA and NHW. Examining individual components of the AL, NHB had specifically a poorer profile in terms of albumin, CRP, glycated hemoglobin, resting heart rate and blood pressure levels compared to NHW (Table 1).

**Table 1:** Baseline characteristics of participants by race/ethnicity group, NHANES III (*n* = 16,573)^a^

	Race/ethnicity				
Selected participant characteristics	NHW	NHB	MA	*p*-value (Design-based F-test)^b^	
Unweighted N	(*N* = 7221) 82.9 %	(*N* = 4846) 11.9 %	(*N* = 4506) 5.2 %	NHB vs. NHW	Mexican-American vs. NHW
*Socio-demographic characteristics*					
Age (years)	45.5 ± 0.5	42.0 ± 0.4	37.6 ± 0.4	<0.001	<0.001
20–49	63.1 ± 1.3	70.8 ± 1.3	80.6 ± 0.9	<0.001	<0.001
50+	36.9 ± 1.3	29.2 ± 1.3	19.4 ± 0.9		
Sex, % male	47.9 ± 0.5	43.7 ± 0.9	52.0 ± 0.7	<0.001	<0.001
Urban/rural area of residence					
Urban	46.5 ± 4.9	59.1 ± 5.6	61.4 ± 6.1	0.011	0.020
Rural	53.5 ± 4.9	40.9 ± 5.6	38.6 ± 6.1		
Household size	2.82 ± 0.04	3.23 ± 0.07	4.33 ± 0.10	<0.001	<0.001
Marital status					
Never married	14.5 ± 0.9	28.2 ± 1.1	17.7 ± 1.1	<0.001	0.008
Married	64.0 ± 1.0	36.1 ± 1.1	60.4 ± 1.3	__	
Divorced	8.0 ± 0.4	11.9 ± 0.8	4.7 ± 0.6	<0.001	0.003
Widowed	7.2 ± 0.4	8.8 ± 0.5	3.5 ± 0.4	<0.001	<0.001
Other	6.3 ± 0.4	15.0 ± 0.6	13.8 ± 1.2	<0.001	<0.001
*Socio-economic status*					
Poverty income ratio	3.28 ± 0.06	2.02 ± 0.06	1.77 ± 0.05	<0.001	<0.001
Education, years	12.7 ± 0.1	11.5 ± 0.1	9.0 ± 0.2	<0.001	<0.001
Insurance status					
Insured	93.8 ± 1.1	90.7 ± 2.0	78.9 ± 4.7	0.034	<0.001
Uninsured	6.2 ± 1.1	9.3 ± 2.0	21.1 ± 4.7		
*Substance abuse*					
Illicit drug use					
Never	63.0 ± 1.4	59.8 ± 1.3	70.6 ± 1.3	0.025	<0.001
Ever	37.0 ± 1.4	40.2 ± 1.3	29.4 ± 1.4		
Alcohol, *g/d*	9.3 ± 0.5	8.8 ± 0.4	8.9 ± 0.6	0.37	0.59
*Nutritional factors*					
1995-HEI total score	64.3 ± 0.3	59.5 ± 0.3	63.9 ± 0.5	<0.001	0.39
MAR total score	74.2 ± 0.3	67.9 ± 0.4	73.7 ± 0.4	<0.001	0.33
*Physical activity 0 = Less, 1 = Same, 2 = more*					
Compare activity for past mo to past yr	0.87 ± 0.01	0.91 ± 0.01	0.88 ± 0.02	0.049	0.74
Active compared with men/women your age	1.13 ± 0.01	1.06 ± 0.01	1.01 ± 0.02	<0.001	<0.001
Active now compared with self 10 year ago	0.61 ± 0.02	0.60 ± 0.01	0.66 ± 0.02	0.50	0.09
*Smoking*					
# cigarettes/day	10.67 ± 0.27	6.68 ± 0.18	5.19 ± 0.15	<0.001	<0.001
Years smoked cigarettes	7.81 ± 0.19	6.16 ± 0.17	5.03 ± 0.17	<0.001	<0.001
*Social support*					
(1) In a typical week, how many times do you talk on the telephone with family, friends, or neighbors?	10.2 ± 0.2	12.8 ± 0.6	6.9 ± 0.3	<0.001	<0.001
(2) How often do you get together with friends or relatives; I mean things like going out together or visiting in each other's homes? (per year)	118.3 ± 2.6	136.9 ± 4.2	108.1 ± 3.8	<0.001	<0.001
(3) About how often do you visit with any of your other neighbors, either in their homes or in your own? (per year)	65.9 ± 3.0	79.3 ± 5.0	54.2 ± 3.4	0.013	0.024
(4) How often do you attend church or religious services? (per year)	30.2 ± 1.1	37.3 ± 2.4	33.3 ± 1.9	0.013	0.13
(5) Altogether, how often do you attend meetings of the clubs or organizations (per year)	13.9 ± 0.6	11.9 ± 0.7	6.6 ± 0.4	0.020	<0.001
*Health-related factors*					
Self-rated health					
Excellent/Very Good	54.6 ± 1.2	38.1 ± 1.4	28.1 ± 0.9	<0.001	<0.001
Good	31.6 ± 0.8	38.1 ± 0.9	40.2 ± 1.1		
Fair/Poor	13.8 ± 0.8	23.7 ± 1.1	31.7 ± 1.2		
Co-morbidity index	0.80 ± 0.02	0.60 ± 0.02	0.37 ± 0.01	<0.001	<0.001
Allostatic load, AL score	1.77 ± 0.04	1.97 ± 0.05	1.84 ± 0.05	0.001	0.30
* AL components*					
Low Albumin	9.6 ± 1.0	19.6 ± 1.5	9.9 ± 0.8	<0.001	0.75
High CRP	27.2 ± 1.3	37.9 ± 1.4	31.9 ± 1.9	<0.001	0.034
High waist-hip ratio	63.4 ± 0.9	57.5 ± 1.3	72.2 ± 0.8	<0.001	<0.001
High total cholesterol	20.2 ± 0.7	17.1 ± 0.7	15.2 ± 1.1	0.004	0.001
Low HDL-C	24.2 ± 0.9	16.0 ± 0.9	26.1 ± 1.3	<0.001	0.23
High glycated hemoglobin	5.1 ± 0.4	10.8 ± 0.6	7.5 ± 0.5	<0.001	0.001
High resting heart rate	6.4 ± 0.4	8.4 ± 0.6	5.7 ± 0.6	0.005	0.33
High systolic blood pressure	15.8 ± 0.8	18.8 ± 0.7	10.5 ± 0.5	0.002	<0.001
High diastolic blood pressure	5.9 ± 0.4	11.1 ± 0.6	5.4 ± 0.4	<0.001	0.42

*Abbreviation*: *HDL-Cholesterol* High-density lipoprotein-Cholesterol, *HEI* Healthy Eating Index, *MA* Mexican-American, *NHANES* National Health and Nutrition Examination Surveys, *NHB* Non-Hispanic Black, *NHW* Non-Hispanic White, *PCA* Principal Components Analysis, *SEM* Standard Error of the Mean, *SEP* Standard Error of the Proportion, *US* United States

^a^Values are weighted means ± SEM or percent ± SEP, taking into account sampling design complexity (PSU and strata), averaged over m = 5 imputations

^b^Design-based F-test took into account design complexity in terms of sampling weights, PSU and stratum. for categorical variables, this was the equivalent of a *χ* ^2^ test of independence restricting the sample first to NHB/NHW, then to Mexican-American/NHW. For continuous variables, it was the equivalent of a Wald test in a linear regression model with the variable being the outcome predicted by race/ethnicity and in which NHW was the referent category to which “NHB” and “Mexican-American” were compared

### Cause of death structure by race/ethnicity

**Fig. 1:**
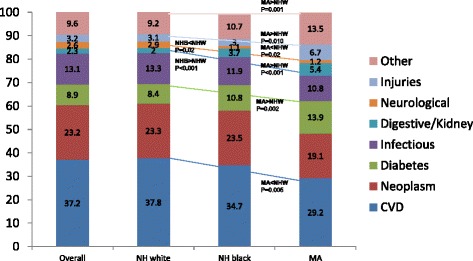
Causes of death structure, overall and by race/ethnicity; NHANES III *

Out of 4207 deaths, around 60.2 % were caused by either cardiovascular disease or a neoplasm, with another 9 % having diabetes as the main contributing factor. Infectious diseases accounted for 13.1 % of deaths, while neurological, digestive/kidney and injuries accounted for 2–3 % of deaths each. The remaining causes, labelled as “Other” accounted for ~9.6 % of deaths, overall. Comparing NHW to NHB, NHB were more likely to have died from digestive/kidney disease than NHW and less likely to have died from neurological disease. When comparing MA to NHW, diabetes, digestive/kidney disease, injuries and “Other” were more common causes of deaths among MA. The reverse was true for CVD and neurological disease (Fig. 1).

### Race/ethnicity and all-cause adult mortality: moderation by sex, age group and poverty status

**Fig. 2:**
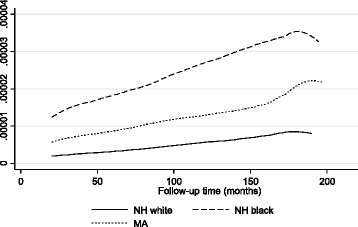
All-cause mortality hazard rates adjusted for age(y), sex and poverty income ratio, by race group; NHANES III

Smoothed all-cause hazard curves by race/ethnicity (adjusted for age, sex and poverty income ratio) were consistently higher among NHBs compared to NHWs and lowest in MA, with an average HR = 1.44, 95 % CI: 1.31–1.58, *p* < 0.001, based on 5 imputations. No difference was noted between MA and NHW in that model (Fig. 2).

Using Cox PH models (Tables 2, 3, 4 and 5), higher all-cause mortality among NHBs vs. NHWs was specific to younger men above poverty (**Model 0,** Table 2: Crude Log_e_(HR) = +0.87, 95 % CI: 0.50;1.24, *p* < 0.001), an association that remained significant after adjustment for age, marital status, urban-rural area of residence and household size (**Model 1,** Table 2). In **Model 2** of Table 3, adjusting further for SES factors (i.e. PIR, education and insurance status), the race/ethnicity associations with all-cause mortality among younger men above poverty was attenuated to Log_e_(HR) = +0.71, 95 % CI:+0.30;1.13, *p* = 0.001, with the fully adjusted model having a similar effect of race on all-cause mortality in that group (Table 5, **model 8**).

**Table 2:** Cox PH of race/ethnicity vs. all-cause mortality: Crude (Model 0)^a^ and socio-demographic factor-adjusted (Model 1)^a^ NHANES III

	Model 0: Crude^b^			Model 1: sociodemographic factor-adjusted^c^		
	Log_e_(HR)	95 % CI	*P*	Log_e_(HR)	95 % CI	*P*
*<50y, women, PIR ≥ 125 %* ^d^	*(N = 3105)*			*(N = 3105)*		
NHB vs. NHW	+0.12	(-0.33;+0.58)	0.60	−0.07	(-0.59;+0.46)	0.81
MA vs. NHW	−0.26	(-0.84;+0.32)	0.37	−0.21	(-0.84;+0.42)	0.43
*<50y, men, PIR ≥ 125 %* ^d^	*(N = 2860)*			*(N = 2860)*		
NHB vs. NHW	+0.87	(+0.50;+1.24)	<0.001	+0.95	(+0.55;+1.34)	<0.001
MA vs. NHW	+0.22	(-0.26;+0.70)	0.37	+0.49	(-0.00;+0.99)	<0.001
*<50y, women, PIR < 125 %* ^d^	*(N = 1728)*			*(N = 1728)*		
NHB vs. NHW	+0.34	(-0.55;+1.24)	0.44	+0.45	(-0.43;+1.32)	0.31
MA vs. NHW	−0.20	(-1.11;+0.70)	0.66	−0.18	(-1.12;+0.76)	0.71
*<50y, men, PIR < 125 %* ^d^	*(N = 1269)*			*(N = 1269)*		
NHB vs. NHW	−0.05	(-0.57;+0.46)	0.55	−0.07	(-0.61;+0.47)	0.80
MA vs. NHW	−0.83	(-1.37;-0.28)	0.003	−0.41	(-0.98;+0.15)	0.15
*≥50y, women, PIR ≥ 125 %* ^d^	*(N = 2420)*			*(N = 2420)*		
NHB vs. NHW	−0.03	(-0.22;+0.17)	0.80	+0.23	(+0.01;+0.46)	0.045
MA vs. NHW	−0.66	(-0.95;-0.38)	<0.001	−0.42	(-0.83;-0.02)	0.040
*≥50y, men, PIR ≥ 125 %* ^d^	*(N = 2443)*			*(N = 2443)*		
NHB vs. NHW	+0.14	(-0.04;+0.32)	0.13	+0.27	(+0.08;+0.46)	0.005
MA vs. NHW	−0.15	(-0.39;+0.08)	0.20	+0.04	(-0.21;+0.29)	0.75
*≥50y, women, PIR < 125 %* ^d^	*(N = 1101)*			*(N = 1101)*		
NHB vs. NHW	−0.10	(-0.32;+0.12)	0.38	−0.00	(-0.27;+0.28)	0.99
MA vs. NHW	−0.61	(-0.88;-0.34)	<0.001	−0.25	(-0.61;+0.10)	0.16
*≥50y, men, PIR < 125 %* ^d^	*(N = 861)*			*(N = 861)*		
NHB vs. NHW	+0.09	(-0.18;0.36)	0.51	+0.09	(-0.16;+0.34)	0.49
MA vs. NHW	−0.56	(-0.87;-0.26)	<0.001	−0.29	(-0.60;+0.03)	0.07

*Abbreviation*: *CI* Confidence Interval, *exp* exponent, *HR* Hazard Ratio, *LCL* Lower confidence limit, *Log*_*e*_ Natural logarithm, *MA* Mexican-American, *NHANES* National Health and Nutrition Examination Surveys, *NHB* Non-Hispanic Black, *NHW* Non-Hispanic White, *PIR* Poverty Income Ratio, *SE* Standard error, *UCL* Upper confidence limit

^a^Values are the natural log of hazard ratios (HR) and 95 % CI with *p*-values, taking into account unequal probability of selection or sampling weights. Statistical significance is inferred from a 95 % CI not crossing the value of zero

^b^Model 0 is crude unadjusted HR

^c^Model 1 adjusted the HR for age, sex, marital status, urban-rural area of residence, and household size, within each age group/sex/poverty status stratum. Note that the point estimate of the HR can be computed as exp(β) where β = Log_e_(HR). The 95 % CI for the HR is computed as exp^(β±1.96*SE(β))^, whereby SE(β) = (UCL_β_ -LCL_β_)/3.92

**Table 3:** Cox PH of race/ethnicity vs. all-cause mortality: further adjustment for SES, substance abuse and dietary factors ^a^, NHANES III

	Model 2: Model 1+ SES factors^b^			Model 3: Further adjusted for substance abuse factors^c^			Model 4: Further adjusted for dietary factors^d^		
	Log_e_(HR)	95 % CI	*P*	Log_e_(HR)	95 % CI	*P*	Log_e_(HR)	95 % CI	*P*
*<50y, women, PIR ≥ 125 %*	*(N = 3105)*			*(N = 3105)*			*(N = 3019)*		
NHB vs. NHW	−0.13	(-0.65;+0.40)	0.64	−0.12	(-0.64;+0.41)	0.66	−0.10	(-0.66;+0.46)	0.73
MA vs. NHW	−0.87	(-1.69;-0.04)	0.040	−0.85	(-1.64;-0.02)	0.044	−0.80	(-1.64;+0.05)	0.06
*<50y, men, PIR ≥ 125 %*	*(N = 2860)*			*(N = 2860)*			*(N = 2773)*		
NHB vs. NHW	+0.71	(+0.30;+1.13)	0.001	+0.71	(+0.29;+1.12)	0.001	+0.70	(+0.28;+1.11)	0.001
MA vs. NHW	+0.06	(-0.50;+0.62)	0.84	+0.04	(-0.53;+0.60)	0.90	−0.06	(-0.65;+0.53)	0.84
*<50y, women, PIR < 125 %*	*(N = 1728)*			*(N = 1728)*			*(N = 1683)*		
NHB vs. NHW	+0.36	(-0.56;+1.27)	0.44	+0.38	(-0.54;+1.31)	0.40	+0.45	(-0.47;+1.37)	0.32
MA vs. NHW	−0.59	(-2.06;+0.87)	0.42	−0.40	(-1.91;+1.10)	0.59	−0.43	(-2.03;+1.17)	0.59
*<50y, men, PIR < 125 %*	*(N = 1269)*			*(N = 1269)*			*(N = 1232)*		
NHB vs. NHW	−0.14	(-0.73;+0.45)	0.64	−0.13	(-0.73;+0.45)	0.65	+0.06	(-0.51;+0.64)	0.84
MA vs. NHW	−0.75	(-1.41;-0.08)	0.027	−0.75	(-1.43;-0.07)	0.031	−0.44	(-1.12;+0.25)	0.21
*≥50y, women, PIR ≥ 125 %*	*(N = 2420)*			*(N = 2420)*			*(N = 2345)*		
NHB vs. NHW	+0.16	(-0.06;+0.38)	0.17	+0.16	(-0.06;+0.38)	0.15	+0.13	(-0.10;+0.36)	0.28
MA vs. NHW	−0.54	(-0.92;-0.15)	0.006	−0.54	(-0.92;-0.15)	0.006	−0.44	(-0.82;-0.07)	0.021
*≥50y, men, PIR ≥ 125 %*	*(N = 2443)*			*(N = 2443)*			*(N = 2339)*		
NHB vs. NHW	+0.19	(-0.00;+0.38)	0.05	+0.19	(-0.01;+0.39)	0.06	+0.06	(-0.15;+0.27)	0.58
MA vs. NHW	−0.08	(-0.35;+0.18)	0.56	−0.08	(-0.36;-0.19)	0.54	−0.07	(-0.35;+0.21)	0.61
*≥50y, women, PIR < 125 %*	*(N = 1101)*			*(N = 1101)*			*(N = 1046)*		
NHB vs. NHW	−0.00	(-0.27;+0.27)	0.98	+0.00	(-0.27;+0.27)	0.99	−0.01	(-0.30;+0.28)	0.93
MA vs. NHW	−0.27	(-0.68;+0.15)	0.21	−0.25	(-0.67;+0.17)	0.24	−0.22	(-0.66;+0.23)	0.34
*≥50y, men, PIR < 125 %*	*(N = 861)*			*(N = 861)*			*(N = 805)*		
NHB vs. NHW	+0.06	(-0.21;+0.32)	0.68	+0.08	(-0.18;+0.34)	0.53	+0.08	(-0.21;+0.38)	0.57
MA vs. NHW	−0.40	(-0.79;-0.02)	0.041	−0.37	(-0.75;+0.02)	0.06	−0.29	(-0.68;+0.10)	0.15

*Abbreviation*: *CI* Confidence Interval, *exp* exponent, *HEI* Healthy Eating Index, *HR* Hazard Ratio, *LCL* Lower confidence limit, *Log*_*e*_ Natural logarithm, *MAR* mean adequacy ratio, *MA* Mexican-American, *NHANES* National Health and Nutrition Examination Surveys, *NHB* Non-Hispanic Black, *NHW* Non-Hispanic White, *PIR* Poverty Income Ratio, *SE* Standard error, *UCL* Upper confidence limit

^a^Values are the natural log of hazard ratios (HR) and 95 % CI with *p*-values, taking into account unequal probability of selection or sampling weights. Statistical significance is inferred from a 95 % CI not crossing the value of zero

^b^Model 2 was Model 1 (Table 2) further adjusted for poverty income ratio, education and health insurance status

^c^Model 3 is Model 2 further adjusted for drug use and alcohol consumption. Model 4 is Model 3 further adjusted for dietary factors (1995-HEI and the MAR total scores). Note that the point estimate of the HR can be computed as exp(β) where β = Log_e_(HR). The 95 % CI for the HR is computed as exp^(β±1.96*SE(β))^, whereby SE(β) = (UCL_β_ -LCL_β_)/3.92

**Table 4:** Cox PH of race/ethnicity vs. all-cause mortality: further adjustment for physical activity, cigarette smoking and social support ^a^, NHANES III

	Model 5: Model 4 further adjusted for physical activity^b^			Model 6: Model 5 further adjusted for cigarette smoking^c^			Model 7: Model 6 further adjusted for social support factors^d^		
	Log_e_(HR)	95 % CI	*P*	Log_e_(HR)	95 % CI	*P*	Log_e_(HR)	95 % CI	*P*
*<50y, women, PIR ≥ 125 %*	*(N = 3019)*			*(N = 3019)*			*(N = 3019)*		
NHB vs. NHW	−0.13	(-0.70;+0.44)	0.65	−0.14	(-0.71;+0.42)	0.63	−0.16	(-0.73;+0.40)	0.57
MA vs. NHW	−0.74	(-1.57;+0.09)	0.08	−0.74	(-1.57;+0.08)	0.08	−0.76	(-1.59;+0.07)	0.07
*<50y, men, PIR ≥ 125 %*	*(N = 2773)*			*(N = 2773)*			*(N = 2773)*		
NHB vs. NHW	+0.72	(+0.31;+1.12)	0.001	+0.83	(+0.37;+1.29)	<0.001	+0.80	(+0.32;+1.28)	<0.001
MA vs. NHW	−0.05	(-0.63;+0.54)	0.88	+0.11	(-0.53;+0.75)	0.74	+0.11	(-0.55;+0.78)	0.73
*<50y, women, PIR < 125 %*	*(N = 1683)*			*(N = 1683)*			*(N = 1683)*		
NHB vs. NHW	+0.43	(-0.48;+1.34)	0.34	+0.43	(-0.48;+1.34)	0.35	+0.49	(-0.42;1.40)	0.29
MA vs. NHW	−0.46	(-2.03;+1.11)	0.56	−0.43	(-1.95;+1.08)	0.57	−0.37	(-1.85;1.10)	0.61
*<50y, men, PIR < 125 %*	*(N = 1232)*			*(N = 1232)*			*(N = 1232)*		
NHB vs. NHW	+0.08	(-0.48;+0.64)	0.79	+0.13	(-0.48;+0.74)	0.68	+0.18	(-0.45;+0.80)	0.58
MA vs. NHW	−0.38	(-1.04;+0.29)	0.26	−0.31	(-1.01;+0.39)	0.39	−0.34	(-1.10;+0.41)	0.37
*≥50y, women, PIR ≥ 125 %*	*(N = 2345)*			*(N = 2345)*			*(N = 2345)*		
NHB vs. NHW	+0.10	(-0.13;+0.33)	0.39	+0.21	(-0.03;+0.44)	0.08	+0.22	(-0.01;+0.45)	0.06
MA vs. NHW	−0.49	(-0.87;-0.12)	0.010	−0.41	(-0.81;-0.01)	0.042	−0.39	(-0.79;+0.00)	0.05
*≥50y, men, PIR ≥ 125 %*	*(N = 2339)*			*(N = 2339)*			*(N = 2339)*		
NHB vs. NHW	+0.09	(-0.13;+0.31)	0.41	+0.16	(-0.06;+0.38)	0.16	+0.17	(-0.05;+0.39)	0.13
MA vs. NHW	−0.05	(-0.33;+0.23)	0.75	+0.04	(-0.25;+0.32)	0.79	+0.05	(-0.23;+0.36)	0.70
*≥50y, women, PIR < 125 %*	*(N = 1046)*			*(N = 1046)*			*(N = 1046)*		
NHB vs. NHW	−0.06	(-0.35;+0.22)	0.65	−0.01	(-0.30;+0.27)	0.93	−0.01	(-0.29;+0.27)	0.95
MA vs. NHW	−0.25	(-0.69;+0.20)	0.27	−0.17	(-0.61;+0.26)	0.43	−0.16	(-0.58;+0.26)	0.95
*≥50y, men, PIR < 125 %*	*(N = 805)*			*(N = 805)*			*(N = 805)*		
NHB vs. NHW	+0.05	(-0.26;+0.36)	0.75	+0.10	(-0.21;+0.41)	0.54	+0.13	(-0.20;0.45)	0.44
MA vs. NHW	−0.32	(-0.72;+0.09)	0.13	−0.24	(-0.68;+0.19)	0.27	−0.25	(-0.69;+0.20)	0.27

*Abbreviation*: *CI* Confidence Interval, *exp* exponent, *HR* Hazard Ratio, *LCL* Lower confidence limit, *Log*_*e*_ Natural logarithm, *MA* Mexican-American, *NHANES* National Health and Nutrition Examination Surveys, *NHB* Non-Hispanic Black, *NHW* Non-Hispanic White, *PIR* Poverty Income Ratio, *SE* Standard error, *UCL* Upper confidence limit

^a^Values are the natural log of hazard ratios (HR) and 95 % CI with *p*-values, taking into account unequal probability of selection or sampling weights. Statistical significance is inferred from a 95 % CI not crossing the value of zero

^b^Model 5 was Model 4 (Table 3) further adjusted for 3 physical activity items

^3^Model 6 is Model 5 further adjusted for cigarette smoking (2 items). Model 7 is Model 6 further adjusted for social support factors (5 items). Note that the point estimate of the HR can be computed as exp(β) where β = Log_e_(HR). The 95 % CI for the HR is computed as exp^(β±1.96*SE(β))^, whereby SE(β) = (UCL_β_ -LCL_β_)/3.92

**Table 5:** Cox PH of race/ethnicity vs. all-cause mortality: further adjustment for health-related factors: full model ^a^, NHANES III

	Model 8: Model 7 further adjusted for health-related factors: Full model^b^		
	Log_e_(HR)	95 % CI	*P*
*<50y, women, PIR ≥ 125 %*	*(N = 3019)*		
NHB vs. NHW	−0.46	(-1.08;+0.15)	0.14
MA vs. NHW	−1.17	(-2.06;-0.29)	0.009
*<50y, men, PIR ≥ 125 %*	*(N = 2,773)*		
NHB vs. NHW	+0.73	(+0.25;+1.22)	0.003
MA vs. NHW	+0.05	(-0.62;+0.72)	0.88
*<50y, women, PIR < 125 %*	*(N = 1,683)*		
NHB vs. NHW	+0.33	(-0.63;+1.29)	0.50
MA vs. NHW	−0.47	(-1.95;+0.99)	0.52
*<50y, men, PIR < 125 %*	*(N = 1,232)*		
NHB vs. NHW	+0.15	(-0.53;+0.84)	0.66
MA vs. NHW	−0.46	(-1.30;+0.38)	0.29
*≥50y, women, PIR ≥ 125 %*	*(N = 2,345)*		
NHB vs. NHW	+0.11	(-0.13;+0.36)	0.36
MA vs. NHW	−0.46	(-0.85;-0.06)	0.024
*≥50y, men, PIR ≥ 125 %*	*(N = 2,339)*		
NHB vs. NHW	+0.12	(-0.11;+0.34)	0.30
MA vs. NHW	+0.03	(-0.26;+0.32)	0.84
*≥50y, women, PIR < 125 %*	*(N = 1,046)*		
NHB vs. NHW	−0.14	(-0.44;+0.16)	0.35
MA vs. NHW	−0.18	(-0.64;+0.27)	0.43
*≥50y, men, PIR < 125 %*	*(N = 805)*		
NHB vs. NHW	+0.05	(-0.28;0.38)	0.75
MA vs. NHW	−0.33	(-0.81;+0.15)	0.18

*Abbreviation*: *CI* Confidence Interval, *exp* exponent, *HR* Hazard Ratio, *LCL* Lower confidence limit, *Log*_*e*_ Natural logarithm, *MA* Mexican-American, *NHANES* National Health and Nutrition Examination Surveys, *NHB* Non-Hispanic Black, *NHW* Non-Hispanic White, *PIR* Poverty Income Ratio, *SE* Standard error, *UCL* Upper confidence limit

^a^Values are the natural log of hazard ratios (HR) and 95 % CI with p-values, taking into account unequal probability of selection or sampling weights. Statistical significance is inferred from a 95 % CI not crossing the value of zero

^b^Model 8 was Model 7 (Table 4) further adjusted for 3 health-related factors (co-morbidity, allostatic load and self-rated health). Note that the point estimate of the HR can be computed as exp(β) where β = Log_e_(HR). The 95 % CI for the HR is computed as exp^(β±1.96*SE(β))^, whereby SE(β) = (UCL_β_ -LCL_β_)/3.92

A lower all-cause mortality rate in MA compared with NHW observed in several age group/sex/poverty strata was retained in the full model only among women above poverty (young and old), (Table 5, **Model 8**). In the crude model (**Model 0**, Table 2), a lower risk of all-cause mortality was found among MA compared to NHW, specifically among men below poverty (both age groups) as well as older women(both poverty status groups). Adjustment for age and socio-demographic factors attenuated the effect appreciably in all these strata. Further adjustment for SES factors, however, pronounced the disparity among women above poverty (both young and old), an effect that was attenuated upon adjustment for dietary factors (**Model 4**, Table 3) as well as in subsequent models. This effect was then markedly pronounced in the final model, particularly among younger women above poverty (**Model 7,** Table 4: -0.76, *p* = 0.07 → **Model 8,** Table 5: -1.17, *p* = 0.009). On the other hand, a higher mortality risk among MA compared with NHW was unveiled upon adjustment for age and other socio-demographic factors, specifically among younger men above poverty (Log_e_(HR): **Model 0,** Table 2: +0.22, *p* = 0.37 → **Model 1,** Table 2: +0.49, *p* < 0.001). This effect, however, was completely explained away by SES factors (**Model 2,** Table 3: +0.06, *p* = 0.84).

### Race/ethnicity and all-cause mortality: individual mediators in the total adult population

Additional file 1: Tables S1 and S2 show a series of generalized SEM models with the outcome being discrete-time hazard of all-cause death, with the main exposure being race/ethnicity, and individual mediators (M) being entered alternatively to explain the total effect of race on hazard rate. Among key findings, PIR, education, dietary quality indices, AL and self-rated health, were all key mediators in the pathway linking race to mortality when comparing NHB to NHW (MP > 10 %). When contrasting hazard rates between MA and NHW, the total effect was not indicative of any racial disparities. Social support factors, drug and alcohol use, as well as insurance status did not act as mediators.

### Race/ethnicity and cause-specific mortality

Table 6 presents findings from competing risk regression models. Age, sex and PIR-adjusted cardiovascular mortality risk was higher in NHBs when compared with NHWs (Log_e_(HR) = +0.22, 95 % CI: +0.07;+0.37, *p* = 0.005), an association that was markedly attenuated in the full model Log_e_(HR) = +0.09, 95 % CI:-0.10;+0.28, *p* = 0.33). In contrast, MA had a lower cardiovascular mortality risk compared with NHWs, particularly in the fully adjusted model.

**Table 6:** Competing risk regression, age, sex and PIR-adjusted vs. full models for direct effect of race on mortality from major causes, NHANES III^a^

	Log_e_(HR)	95 % CI	*P*
*Cardiovascular mortality*			
*Age, sex, PIR-adjusted model* ^b^			
NHB vs. NHW	+0.22	(+0.07;+0.37)	0.005
MA vs. NHW	−0.21	(-0.42;0.00)	0.05
*Full model* ^c^			
NHB vs. NHW	+0.09	(-0.10;0.28)	0.33
MA vs. NHW	−0.31	(-0.58;-0.04)	0.024
*Neoplasms*			
*Age, sex, PIR-adjusted model* ^b^			
NHB vs. NHW	+0.24	(+0.05;+0.44)	0.014
Mexican-American vs. NHW	−0.33	(-0.60;-0.05)	0.021
*Full model* ^c^			
NHB vs. NHW	+0.35	(+0.01;+0.68)	0.044
MA vs. NHW	+0.21	(-0.23;0.64)	0.35

*Abbreviation*: *CI* Confidence Interval, *HEI* Healthy Eating Index, *HR* Hazard Ratio, *MA* Mexican-American, *MAR* mean adequacy ratio, *NHANES* National Health and Nutrition Examination Surveys, *NHB* Non-Hispanic Black, *NHW* Non-Hispanic White, *PIR* Poverty Income Ratio

^a^Values are the natural log of hazard ratios (HR) and 95 % CI with p-values, taking into account unequal probability of selection or sampling weights. Statistical significance is inferred from a 95 % CI not crossing the value of zero

^b^The age, sex and PIR adjusted model is presented here to be contrasted with the full model

^c^The full model adjusted for the same vector of covariates as in Model 8 (Table 5). Note that the point estimate of the HR can be computed as exp(β) where β = Log_e_(HR). The 95 % CI for the HR is computed as exp^(β±1.96*SE(β))^, whereby SE(β) = (UCL_β_ -LCL_β_)/3.92

Moreover, NHBs had a greater neoplasm-related mortality risk compared to NHWs in both the age, sex and PIR-adjusted and the full model. A lower risk of neoplasm-related death among MAs when compared with NHWs was completely explained away in the full model by factors beyond age, sex and PIR.

## Discussion

Using nationally representative data on US adults, we examined all-cause and cause-specific mortality disparities by race/ethnicity, mediation through key factors and moderation by age (20–49 vs. 50+), sex and poverty status. Among key findings, age, sex and poverty income ratio-adjusted hazard rates were higher among NHBs vs. NHWs. Within the above-poverty young men stratum where this association was the strongest, the socio-demographic-adjusted HR = 2.59, *p* < 0.001 was only partially attenuated by SES and other factors (full model HR = 2.08, *p* = 0.003). Income, education, diet quality, allostatic load and self-rated health, were among key mediators explaining NHB *vs*. NHW disparity in mortality. The Hispanic paradox was observed consistently among women above poverty (young and old). NHBs had higher CVD-related mortality risk compared to NHW which was explained by factors beyond SES. Those factors did not explain excess risk among NHBs for neoplasm-related death (fully adjusted HR = 1.41, 95 % CI: 1.02–2.75, *p* = 0.044). Moreover, those factors explained the lower risk of neoplasm-related death among MAs compared to NHW, while CVD-related mortality risk became lower among MAs compared to NHWs upon multivariate adjustment.

Race disparities in all-cause and cause-specific mortality rates, including death from cardiovascular disease and cancer, among U.S. adults have been previously reported, whereby Blacks or African Americans experienced consistently higher mortality rates compared to Whites [1, 24–26]. Several mediating and moderating factors have been examined in an attempt to explain these race disparities, including the moderating effects of gender, [27] age [4, 28, 29] – described as “Black-White mortality crossover” – and obesity, [30] the mediating [24, 29, 31–33] or moderating effects [4, 11, 25, 34, 35] of social factors, including poverty, culture and social injustice, [24] socioeconomic position, [25] socioeconomic status, [32, 35] social class, [36] education, [4] income, [4, 33, 34, 36] perceived stress, [31] health behaviors [31, 32] and health insurance [32]. Previous studies that examined these mediating and moderating effects were based on surveillance or large cohort data, including vital statistics, [1, 26] the National Health Interview Study, [28, 31, 36] the National Cancer Institute’s Surveillance, Epidemiology, and End Results program, [37] the Southern Community Cohort Study, [30, 35] the Health and Retirement Study, [32] the Multiple Risk Factor Intervention Trial, [33] the Americans’ Changing Lives Study [29].

A consistent finding from previous studies is that socioeconomic factors can moderate the effect of race on risk of death [4, 11, 25]. In addition, socio-economic status and other factors can act in mediating racial disparities in all-cause mortality [31, 32]. In a recent study, Krueger and colleagues used 1990 National Health Interview data involving 38,891 US adults and found distinct mediating effects of socioeconomic status, smoking status, physical activity, perceived stress, sleep duration and alcohol consumption on the relationship between race and all-cause mortality [31]. Similarly, analysis of the 1992–1998 Health and Retirement Study found distinct mediating effects of socioeconomic status, health behaviors and health insurance as mediators of the race disparities in all-cause mortality rates [32].

We find that NHBs had a higher rate of CVD mortality compared to NHWs, which is in accord with previous investigations [25, 33]. Our findings also suggest that by factors beyond SES mediated this association. Other variables may be important in explaining the higher CVD mortality in NHBs. Jones-Webb et al. found that neighborhood socioeconomic status moderated associations between race and CVD mortality among older men; older NHBs living in impoverished neighborhoods had a higher rate of CVD mortality, compared to older NHWs living in similar conditions [25]. Unsurprisingly, increased prevalence of CVD risk factors in NHBs may explain the higher prevalence of CVD mortality in this group [33].

Neoplasm-related death rates among NHB have remained high or have increased over time in certain instances [38]. Racial/Ethnic differences in neoplasm-related mortality can result from a combination of factors including smoking, nutrition, access to preventive, diagnostic, therapeutic, screening services and aggressiveness of treatment [38]. Modifying those factors could potentially prevent over half of cancer deaths and eliminate most racial/ethnic disparities [38]. Specifically, racial differences in breast cancer survival prevailed even after controlling for disease stage and known tumor characteristics, reflecting the potential mediating effects of social determinants beyond the biological, genetic and environmental factors, including the barriers of poverty (e.g. lack of a primary care physician, geographical access to care, competing survival priorities, burden of comorbidities, health insurance status, lack of information and knowledge, risk-promoting lifestyles and provider/system-level factors), culture (spirituality, perceived susceptibility to breast cancer, cultural beliefs and attitudes, and medical mistrust) and social injustice (racial prejudice and injustice) [24]. Menashe et al. indicated that the rate of decline in breast cancer mortality was slower among NH black women compared to White women, while age-specific incidence rate in black women was lower among blacks. Thus, the widening disparity in breast cancer mortality could not be explained by a higher incidence rate between 1990 and 2004 [37]. Thorpe et. al. showed that there was about 70 % excess risk of cancer mortality among Blacks compared to Whites, with socio-economic status, health insurance, psychosocial factors, behavioral factors and self-rated health accounting for 30 %, 18 %, 1 %, 17 % and 8 % of this excess risk [12]. Our study indicated that there was a 41 % excess risk of neoplasm-related death among NHBs compared to NHWs, which were not explained by SES, lifestyle or health-related factors.

In terms of diabetes-related mortality, studies have suggested that NHB have more than double the risk compared to NHW [12]. Using national death files and census data, for the 50 most populous US cities, that age-adjusted rate ratios of mortality from diabetes were higher in NHB compared to NHW in 39 of 41 cities, ranging from 1.57 (95 % CI: 1.33–1.86) in Baltimore to 3.78 (95 % CI: 2.84–5.02) in Washington, DC. Poverty alone explained 58.5 % of the NHB/NHW disparity in diabetes-related mortality and segregation explained 72.6 % of the disparity. However, those mediating effects of poverty and segregation varied widely across US cities [39]. Between 1994 and 2001, the annual rate of newly diagnosed elderly individuals with diabetes increased by 36.9 %, overall with Hispanics having the greatest increase at 55 % [40]. Our study indicated that MA had indeed a greater share of deaths attributed to diabetes compared to NHWs.

The Hispanic paradox, a consistently observed phenomenon, [9] occurs when mortality rates, specifically cardiovascular [6] and smoking-related mortality [5] among US Hispanics is similar or lower to NHWs’ rates, despite lower SES among Hispanics. Hunt et al. reported the age and sex-adjusted HR for all-cause mortality of US-born MAs vs. NHWs as 1.66 (95 % CI 1.15–2.40), while Mexico-born MAs vs. NHWs as 1.14 (95 % CI 0.63–2.06), [41] suggesting that “acculturation” in young MA may be a multifactorial covariate that is inadequately represented in large study sets such as NHANES III, which is sampling for a “paradoxically healthy” new immigrant population, rather than a truly representative sample of young Mexicans as a whole [42]. Generally, mortality rate differences between MA and NHW are greater among older age groups. Suggested mechanisms behind this paradox include less acculturation to the US resulting in better health, healthy migrant bias, and death records’ misreporting of ethnicity or missing records upon return to country of origin (“salmon bias”) [43]. Previous studies show that diet was healthier and smoking level was lower among Hispanics compared to non-Hispanics, which may partly explain their lower mortality rates [7, 8]. These findings are not universal, with studies in San Antonio [44, 45] and Corpus Christi [46] refuting the apparent paradox. Our findings support the Hispanic paradox mainly for cardiovascular mortality, which concurs with a recent meta-analysis [6]. It has been suggested that increased fruit and legume consumption among this group may have a protective effect [6]. Country of birth may be an important consideration; data from the San Antonio Heart Study show that diabetic MAs born in the US have higher rates of CVD mortality, compared to NHWs, while risk for CVD mortality was similar between diabetic US-born MAs and NHWs [41]. The findings also support the perplexing disassociation of several common risk factors with cardiovascular disease mortality in US Hispanic populations. MAs in our study had both lower income and a lower mean education years, when compared with NHWs. They also had a higher waist-hip-ratio and glycated hemoglobin levels, as well as lower access to health insurance. In contrast, MA were less likely to smoke and had a comparable diet quality to NHW. Smoking behavior differentials have accounted for >50 % life expectancy variability between Hispanics and non-Hispanics at age 50y [7]. Acculturation may influence our findings as only 51 % of MA in our sample were US-born.

Our study has several strengths. First, to our knowledge, it among few nationally representative studies testing associations between race/ethnicity and all-cause and cause-specific mortality in the adult US population by systematically examining effects within sex, age and poverty status and investigating potential mediators for all-cause and cause-specific mortality. Second, its large sample size allowed testing associations with mortality from homogeneous groups of causes. Competing risk, selection bias, missing data, unequal probability of sampling and design complexity were all addressed in our analyses. Some limitations include residual confounding, measurement error in covariates, particularly self-reported potential mediators (e.g. co-morbid conditions), and misclassification error of underlying and contributing causes of death.

## Conclusion

In sum, racial/ethnic disparities in all-cause and cause-specific mortality (particularly cardiovascular and neoplasms) were partly explained by socio-demographic, SES, health-related and dietary factors, and differentially by age, sex and poverty strata. More studies are needed to uncover neighborhood-level and individual-level psychosocial factors mediating the effect of racial disparities on all-cause and cause-specific mortality among US adults.

## Additional file

Additional file 1: Table S1. Total and direct effects of race on all cause mortality and effects mediated through socio-economic, lifestyle and social support factors, NHANES III. **Table S2.** Total and direct effects of race on all-cause mortality and effects mediated through health-related factors, NHANES III. (DOCX 54 kb)

## Abbreviations

AL: Allostatic load
CDC: Centers for disease control and prevention
CI: Confidence interval
DBP: Diastolic blood pressure
HDL-C: High-density lipoprotein-cholesterol
HEI: Healthy eating index
HR: Hazard ratio
HR: Hazard ratio
ICD-10: International classification of diseases
MA: Mexican-American
MAR: Mean adequacy ratio
MEC: Mobile examination center
MP: Mediation proportion
NCHS: National center for health statistics
NDI: National death index
NHANES: National health and nutrition examination surveys
NHB: Non-hispanic black
NHW: Non-hispanic white
SBP: Systolic blood pressure
TC: Total cholesterol
TG: Triglycerides
US: United States
USDA: US department of agriculture
