# Supplementary material for: Racial disparities in adult all-cause and cause-specific mortality among us adults: mediating and moderating factors
Source: BMC Public Health. 2016 Oct 22;16:1113. doi: 10.1186/s12889-016-3744-z (PMC5075398; doi:10.1186/s12889-016-3744-z)
Supplement: Additional file 1: Table S1. — Total and direct effects of race on all cause mortality and effects mediated through socio-economic, lifestyle and social support factors, NHANES III. Table S2. Total and direct effects of race on all-cause mortality and effects mediated through health-related factors, NHANES III. (DOCX 54 kb) [file 12889_2016_3744_MOESM1_ESM.docx]

**APPENDIX I: 1995-HEI, MAR and AL**

**MAR scores**

RDAs of 16 vitamins and minerals were used to determine the nutrient adequacy ratio (NAR), using the following formula: NAR = [Subject’s daily intake of nutrient] / [RDA of nutrient]. An adjustment of an additional 35 mg Vitamin C must be applied to the RDA for participants who were current smokers.

The NAR of each nutrient is converted to a percent, and percentages greater than 100 are truncated to 100. The total quality of the diet is then calculated from the NARs to form a mean adequacy ratio (MAR) using the following formula: MAR = [Sum of all 16 nutrient NARs]/16. The NAR and MAR for each day were calculated and then averaged over the two days.

**Table I. 1.** Recommended Dietary Allowance (RDA) Values for Nutrients accessed from USDA DRI Tables to MAR Score

| **Nutrient** | **Men**  **19-30yrs** | **Men**  **31-50yrs** | **Men**  **51-70 yrs** | **Women**  **19-30yrs** | **Women**  **31-50yrs** | **Women**   - 1. **yrs** |
| --- | --- | --- | --- | --- | --- | --- |
| Vitamin A | 900 ug/day | 900 ug/day | 900 ug/day | 700 ug/day | 700 ug/day | 700 ug/day |
| Vitamin C | 90 mg/day | 90 mg/day | 90 mg/day | 75 mg/day | 75 mg/day | 75 mg/day |
| **Vitamin C for smokers** | 125 mg/day | 125 mg/day | 125 mg/day | 110 mg/day | 110 mg/day | 110 mg/day |
| Vitamin D | 15 ug/day | 15 ug/day | 15 ug/day | 15 ug/day | 15 ug/day | 15 ug/day |
| Vitamin E | 15 mg/day | 15 mg/day | 15 mg/day | 15 mg/day | 15 mg/day | 15 mg/day |
| Vitamin B6 | 1.3 mg/day | 1.3 mg/day | 1.7 mg/day | 1.3 mg/day | 1.3 mg/day | 1.5 mg/day |
| Vitamin B12 | 2.4 ug/day | 2.4 ug/day | 2.4 ug/day | 2.4 ug/day | 2.4 ug/day | 2.4 ug/day |
| Thiamin | 1.2 mg/day | 1.2 mg/day | 1.2 mg/day | 1.1 mg/day | 1.1 mg/day | 1.1 mg/day |
| Riboflavin | 1.3 mg/ day | 1.3 mg/ day | 1.3 mg/day | 1.1 mg/day | 1.1 mg/day | 1.1 mg/day |
| Niacin | 16 mg day | 16 mg day | 16 mg/day | 14 mg/day | 14 mg/day | 14 mg/day |
| Folate | 400 ug/day | 400 ug/day | 400 ug/day | 400 ug/day | 400 ug/day | 400 ug/day |
| Iron | 8 mg/day | 8 mg/day | 8 mg/day | 18 mg/day | 18 mg/day | 8 mg/day |
| Copper | 900 ug/day | 900 ug/day | 900 ug/day | 900 ug/day | 900 ug/day | 900 ug/day |
| Zinc | 11 mg/day | 11 mg/day | 11 mg/day | 8 mg/day | 8 mg/day | 8 mg/day |
| Calcium | 1,000 mg/day | 1,000 mg/day | 1,000 mg/day | 1,000 mg/day | 1,000 mg/day | 1,200 mg/day |
| Magnesium | 400 mg/day | 420 mg/day | 420 mg/day | 310 mg/day | 320 mg/day | 320 mg/day |
| Phosphorous | 700 mg/day | 700 mg/day | 700 mg/day | 700 mg/day | 700 mg/day | 700 mg/day |

ug= micrograms; mg= milligrams; g=grams

<http://www.nal.usda.gov/fnic/DRI/DRI_Tables/RDA_AI_vitamins_elements.pdf>

<http://ods.od.nih.gov/factsheets/VitaminC-HealthProfessional/>

**Allostatic Load (AL)**

A total AL score was computed using a method described in a previous study.[[1](#_ENREF_1)] AL total score sums up cardiovascular (systolic and diastolic blood pressure, pulse rate), metabolic (total cholesterol, HDLcholesterol, glycosylated Hb, sex-specific waist-to-hip ratio) and inflammatory (albumin and C-reactive protein (CRP)) risk indicators. Clinical criteria summarized in Table I.2 were used to obtain risk indicators which were summed with equal weighting to compute total AL score (range: 0-9).

Total cholesterol (mg/dl), HDL-cholesterol (mg/dl), CRP (mg/dl), albumin (g/dl) and glycosylated hemoglobin (%) were measured by contract laboratories using reference analytical methods (See Laboratory Procedures for NHANES III).[[2](#_ENREF_2)] Using standard protocols, waist-to-hip ratio, radial pulse (beats/min), and systolic and diastolic blood pressure (mmHg) were measured by trained examiners. Specifically, blood pressure was measured using a mercury sphygmomanometer [[2](#_ENREF_2)] The arithmetic mean of three systolic and diastolic pressures was used in analysis.

**Table I.2 Allostatic load indicator criteria[**[**1**](#_ENREF_1)**]**

|  | High-risk clinical |
| --- | --- |
| Albumin (g/dL) | < 3.8 [[3](#_ENREF_3)] |
| C-reactive protein (mg/dL) | ≥ 0.3 [[4](#_ENREF_4)] |
| Waist:Hip | >0.9 for men; > 0.85 for women [[5](#_ENREF_5)] |
| Total cholesterol (mg/dL) | ≥240[[6](#_ENREF_6)] |
| HDL (mg/dL) | <40[[6](#_ENREF_6)] |
| Glycated hemoglobin (%) | ≥6.4[[7](#_ENREF_7), [8](#_ENREF_8)] |
| Resting heart rate (beat/min) | ≥90[[9](#_ENREF_9)] |
| Systolic BP | ≥140[[10](#_ENREF_10)] |
| Diastolic BP | ≥90[[10](#_ENREF_10)] |

**References**

1. Seeman T, Merkin SS, Crimmins E, Koretz B, Charette S, Karlamangla A: **Education, income and ethnic differences in cumulative biological risk profiles in a national sample of US adults: NHANES III (1988-1994)**. *Social science & medicine* 2008, **66**(1):72-87.

2. Gunter EW, Lewis, B. G., Koncikowski, S. M. : **Laboratory Procedures used for the Third National Health and Nutrition Examination Survey (NHANES III), 1988–1994**. In*.* Edited by US Department of Health and Human Services CfDCaP, Hyattsville, MD; 1996.

3. Visser M, Kritchevsky SB, Newman AB, Goodpaster BH, Tylavsky FA, Nevitt MC, Harris TB: **Lower serum albumin concentration and change in muscle mass: the Health, Aging and Body Composition Study**. *Am J Clin Nutr* 2005, **82**(3):531-537.

4. Ridker PM: **Cardiology Patient Page. C-reactive protein: a simple test to help predict risk of heart attack and stroke**. *Circulation* 2003, **108**(12):e81-85.

5. Alberti KG, Zimmet PZ: **Definition, diagnosis and classification of diabetes mellitus and its complications. Part 1: diagnosis and classification of diabetes mellitus provisional report of a WHO consultation**. *Diabetic medicine : a journal of the British Diabetic Association* 1998, **15**(7):539-553.

6. Expert Panel on Detection E, Treatment of High Blood Cholesterol in A: **Executive Summary of The Third Report of The National Cholesterol Education Program (NCEP) Expert Panel on Detection, Evaluation, And Treatment of High Blood Cholesterol In Adults (Adult Treatment Panel III)**. *Jama* 2001, **285**(19):2486-2497.

7. Golden S, Boulware LE, Berkenblit G, Brancati F, Chander G, Marinopoulos S, Paasche-Orlow M, Powe N, Rami T: **Use of glycated hemoglobin and microalbuminuria in the monitoring of diabetes mellitus**. *Evidence report/technology assessment* 2003(84):1-6.

8. Osei K, Rhinesmith S, Gaillard T, Schuster D: **Is glycosylated hemoglobin A1c a surrogate for metabolic syndrome in nondiabetic, first-degree relatives of African-American patients with type 2 diabetes?** *The Journal of clinical endocrinology and metabolism* 2003, **88**(10):4596-4601.

9. Seccareccia F, Pannozzo F, Dima F, Minoprio A, Menditto A, Lo Noce C, Giampaoli S, Malattie Cardiovascolari Aterosclerotiche Istituto Superiore di Sanita P: **Heart rate as a predictor of mortality: the MATISS project**. *American journal of public health* 2001, **91**(8):1258-1263.

10. Lenfant C, Chobanian AV, Jones DW, Roccella EJ, Joint National Committee on the Prevention DE, Treatment of High Blood P: **Seventh report of the Joint National Committee on the Prevention, Detection, Evaluation, and Treatment of High Blood Pressure (JNC 7): resetting the hypertension sails**. *Hypertension* 2003, **41**(6):1178-1179.

**Table S1.** Total and direct effects of race on all-cause mortality and effects mediated through socio-economic, lifestyle and social support factors, NHANES III

|  |  | NHB vs. NHW |  | Mexican-American vs. NHW |  |  |
| --- | --- | --- | --- | --- | --- | --- |
| **M=PIR** |  | (N=130,890)^1^ |  | (N=129,794)^1^ |  |  |
| Direct effect of race: Race 🡪 Hazard rate^2^ |  | **+0.195±0.057**** |  | -0.07±0.04~ |  |  |
| Race 🡪 PIR |  | **-0.948±0.012***** |  | **-0.571±0.006***** |  |  |
| PIR 🡪 Hazard rate^1^ |  | **-0.138±0.018***** |  | **-0.137±0.019***** |  |  |
| Indirect effect: Race 🡪 PIR🡪Hazard rate |  | **+0.131±0.017***** |  | **+0.078±0.011***** |  |  |
| Mediation proportion, % |  | **+40.2±8.6***** |  | >100(ns) |  |  |
| **M=EDUC** |  | (N=141,313)^1^ |  | (N=141,756)^1^ |  |  |
| Direct effect of race: Race 🡪 Hazard rate^2^ |  | **+0.225±0.055***** |  | **-0.110±0.044*** |  |  |
| Race 🡪 EDUC |  | **-1.019±0.018***** |  | **-1.851±0.012***** |  |  |
| EDUC 🡪 Hazard rate^1^ |  | **-0.050±0.007***** |  | **-0.051±0.008***** |  |  |
| Indirect effect: Race 🡪 EDUC🡪Hazard rate |  | **+0.052±0.007***** |  | **+0.094±0.015***** |  |  |
| Mediation proportion, % |  | **+18.7±4.9***** |  | >100(ns) |  |  |
| **M=INSURED** |  | (N=141,885)^1^ |  | (N=142,490)^1^ |  |  |
| Direct effect of race: Race 🡪 Hazard rate^2^ |  | **+0.330±0.051***** |  | +0.003±0.037 |  |  |
| Race 🡪 INSURED |  | -0.005±0.027 |  | **-0.535±0.012***** |  |  |
| INSURED 🡪 Hazard rate^2^ |  | **-0.474±0.135***** |  | **-0.504±0.147***** |  |  |
| Indirect effect: Race 🡪 INSURED🡪Hazard rate |  | +0.002±0.013 |  | **+0.27±0.08***** |  |  |
| Mediation proportion, % |  | +0.64±3.89 |  | **+99.00±13.5***** |  |  |
| **M=DRUGS** |  | (N=99,809)^1^ |  | (N=100,076)^1^ |  |  |
| Direct effect of race: Race 🡪 Hazard rate^2^ |  | **+0.508±0.102***** |  | +0.052±0.067 |  |  |
| Race 🡪 DRUGS |  | **-0.125±0.017***** |  | **-0.540±0.011***** |  |  |
| DRUGS 🡪 Hazard rate^2^ |  | +0.095±0.134 |  | +0.070±0.158 |  |  |
| Indirect effect: Race 🡪 DRUGS🡪Hazard rate |  | -0.01±0.02 |  | -0.038±0.085 |  |  |
| Mediation proportion, % |  | -2.41±3.51 |  | >100(ns) |  |  |
| **M=ALCOHOL** |  | (N=137,242)^1^ |  | (N=137,858)^1^ |  |  |
| Direct effect of race: Race 🡪 Hazard rate^2^ |  | **+0.338±0.053***** |  | +0.043±0.037 |  |  |
| Race 🡪 ALCOHOL |  | **-1.283±0.178***** |  | **-0.685±0.100***** |  |  |
| ALCOHOL 🡪 Hazard rate^2^ |  | -0.000±0.002 |  | -0.001±0.002 |  |  |
| Indirect effect: Race 🡪 ALCOHOL🡪Hazard rate |  | +0.000±0.002 |  | +0.000±0.001 |  |  |
| Mediation proportion, % |  | +0.14±0.68 |  | +1.02±3.24 |  |  |
| **M=HEI** |  | (N=137,590)^1^ |  | (N=138,334)^1^ |  |  |
| Direct effect of race: Race 🡪 Hazard rate^2^ |  | **+0.289±0.054***** |  | +0.040±0.036 |  |  |
| Race 🡪 HEI |  | **-4.05±0.09***** |  | **+0.694±0.048***** |  |  |
| HEI 🡪 Hazard rate^2^ |  | **-0.010±0.002***** |  | **-0.011±0.002***** |  |  |
| Indirect effect: Race 🡪 HEI🡪Hazard rate |  | **+0.042±0.007***** |  | **-0.008±0.001***** |  |  |
| Mediation proportion, % |  | **+12.6±3.1***** |  | -23.6±27.2 |  |  |
| **M=MAR** |  | (N=137,590)^1^ |  | (N=138,334)^1^ |  |  |
| Direct effect of race: Race 🡪 Hazard rate^2^ |  | **+0.266±0.055***** |  | +0.023±0.037 |  |  |
| Race 🡪 MAR |  | **-5.942±0.117***** |  | **-0.359±0.063***** |  |  |
| MAR 🡪 Hazard rate^2^ |  | **-0.009±0.002***** |  | **-0.010±0.002***** |  |  |
| Indirect effect: Race 🡪 MAR🡪Hazard rate |  | **+0.052±0.009***** |  | **+0.004±0.001***** |  |  |
| Mediation proportion, % |  | **+16.3±4.3***** |  | 13.1±18.2 |  |  |
| **M=PA item 1** |  | (N=141,713)^1^ |  | (N=142,341)^1^ |  |  |
| Direct effect of race: Race 🡪 Hazard rate^2^ |  | **+0.440±0.047***** |  | **+0.073±0.034*** |  |  |
| Race 🡪 PA item 1 |  | **+0.050±0.005***** |  | +0.002±0.003 |  |  |
| PA item 1 🡪 Hazard rate^2^ |  | -0.030±0.045 |  | -0.048±0.049 |  |  |
| Indirect effect: Race 🡪 PA item 1 🡪Hazard rate |  | -0.002±0.002 |  | -0.000±0.000 |  |  |
| Mediation proportion, % |  | -0.34±0.52 |  | -0.111±0.215 |  |  |
| **M=PA item 2** |  | (N=139,315)^1^ |  | (N=139,593)^1^ |  |  |
| Direct effect of race: Race 🡪 Hazard rate^2^ |  | **+0.272±0.052***** |  | +0.002±0.036 |  |  |
| Race 🡪 PA item 2 |  | **-0.0180±0.005**** |  | **-0.027±0.003***** |  |  |
| PA item 2 🡪 Hazard rate^2^ |  | **-0.423±0.036***** |  | **-0.443±0.039***** |  |  |
| Indirect effect: Race 🡪 PA item 2 🡪Hazard rate |  | **+0.008±0.002***** |  | **+0.012±0.002***** |  |  |
| Mediation proportion, % |  | **+2.72±0.98**** |  | 88.00±233.6 |  |  |
| **M=PA item 3** |  | (N=112,502)^1^ |  | (N=109,697)^1^ |  |  |
| Direct effect of race: Race 🡪 Hazard rate^2^ |  | **+0.342±0.052***** |  | +0.016±0.037 |  |  |
| Race 🡪 PA item 3 |  | **-0.065±0.006***** |  | -0.007±0.003~ |  |  |
| PA item 3 🡪 Hazard rate^2^ |  | **-0.227±0.044***** |  | **-0.235±0.051***** |  |  |
| Indirect effect: Race 🡪 PA item 3 🡪Hazard rate |  | **+0.015±0.003***** |  | +0.002±0.001 |  |  |
| Mediation proportion, % |  | **+4.11±1.04***** |  | +8.93±20.00 |  |  |
| **M=SMOKE item 1** |  | (N=105,838)^1^ |  | (N=102,138)^1^ |  |  |
| Direct effect of race: Race 🡪 Hazard rate^2^ |  | **+0.421±0.065***** |  | +0.077±0.046~ |  |  |
| Race 🡪 SMOKE item 1 |  | **-3.316±0.085***** |  | **-3.36±0.05***** |  |  |
| SMOKE item 1 🡪 Hazard rate^2^ |  | **+0.025±0.002***** |  | +0.025±0.003 |  |  |
| Indirect effect: Race 🡪 SMOKE item 1🡪Hazard rate |  | **-0.084±0.008***** |  | **-0.085±0.009***** |  |  |
| Mediation proportion, % |  | **-24.87±5.09***** |  | >100(ns) |  |  |
| **M=SMOKE item 2** |  | (N=106,087)^1^ |  | (N=103,587)^1^ |  |  |
| Direct effect of race: Race 🡪 Hazard rate^2^ |  | **+0.362±0.064***** |  | +0.009±0.046 |  |  |
| Race 🡪 SMOKE item 2 |  | **-0.400±0.057***** |  | **-0.913±0.030***** |  |  |
| SMOKE item 2 🡪 Hazard rate^2^ |  | **+0.021±0.002***** |  | **+0.020±0.002***** |  |  |
| Indirect effect: Race 🡪 SMOKE item 2🡪Hazard rate |  | **-0.008±0.001***** |  | **-0.018±0.002***** |  |  |
| Mediation proportion, % |  | **-2.32±0.59***** |  | >100(ns) |  |  |
| **M=SS item 1** |  | (N=135,252)^1^ |  | (N=134,831)^1^ |  |  |
| Direct effect of race: Race 🡪 Hazard rate^2^ |  | **+0.325±0.054***** |  | +0.023±0.037 |  |  |
| Race 🡪 SS item 1 |  | **+1.928±0.132***** |  | **-1.527±0.055***** |  |  |
| SS item 1 🡪 Hazard rate^2^ |  | -0.002±0.002 |  | -0.001±0.003 |  |  |
| Indirect effect: Race 🡪 SS item 1 🡪Hazard rate |  | -0.003±0.005 |  | +0.003±0.004 |  |  |
| Mediation proportion, % |  | -1.07±1.52 |  | +10.65±22.50 |  |  |
| **M=SS item 2** |  | (N=141,792)^1^ |  | (N=142,370)^1^ |  |  |
| Direct effect of race: Race 🡪 Hazard rate^2^ |  | **+0.329±0.052***** |  | +0.025±0.034 |  |  |
| Race 🡪 SS item 2 |  | **+6.05±1.03***** |  | **-3.00±0.509***** |  |  |
| SS item 2 🡪 Hazard rate^2^ |  | +0.000±0.000 |  | +0.000±0.000 |  |  |
| Indirect effect: Race 🡪 SS item 2 🡪Hazard rate |  | +0.001±0.001 |  | -0.000±0.000 |  |  |
| Mediation proportion, % |  | +0.20±0.20 |  | -1.333±2.370 |  |  |
| **M=SS item 3** |  | (N=141,735)^1^ |  | (N=142,414)^1^ |  |  |
| Direct effect of race: Race 🡪 Hazard rate^2^ |  | **+0.328±0.051***** |  | +0.027±0.036 |  |  |
| Race 🡪 SS item 3 |  | **+11.978±1.034***** |  | **-2.860±0.476***** |  |  |
| SS item 3 🡪 Hazard rate^2^ |  | +0.000±0.00 |  | +0.000±0.000 |  |  |
| Indirect effect: Race 🡪 SS item 3 🡪Hazard rate |  | +0.001±0.001 |  | -0.000±0.000 |  |  |
| Mediation proportion, % |  | +0.31±0.35 |  | -0.95±1.65 |  |  |
| **M=SS item 4** |  | (N=141,714)^1^ |  | (N=142,425)^1^ |  |  |
| Direct effect of race: Race 🡪 Hazard rate^2^ |  | **+0.345±0.051***** |  | +0.031±0.034 |  |  |
| Race 🡪 SS item 4 |  | **+9.207±0.037***** |  | **+3.069±0.287***** |  |  |
| SS item 4 🡪 Hazard rate^2^ |  | **-0.003±0.001***** |  | **-0.003±0.001***** |  |  |
| Indirect effect: Race 🡪 SS item 4 🡪Hazard rate |  | **-0.026±0.005***** |  | **-0.009±0.002***** |  |  |
| Mediation proportion, % |  | **-8.37±2.11***** |  | -39.9±66.8 |  |  |
| **M=SS item 5** |  | (N=141,732)^1^ |  | (N=142,382)^1^ |  |  |
| Direct effect of race: Race 🡪 Hazard rate^2^ |  | **+0.326±0.051***** |  | +0.020±0.036 |  |  |
| Race 🡪 SS item 5 |  | **-2.062±0.281***** |  | **-3.594±0.136***** |  |  |
| SS item 5 🡪 Hazard rate^2^ |  | -0.001±0.0001 |  | -0.002±0.001 |  |  |
| Indirect effect: Race 🡪 SS item 4 🡪Hazard rate |  | +0.003±0.002 |  | +0.005±0.004 |  |  |
| Mediation proportion, % |  | +0.89±0.61 |  | 20.1±32.6 |  |  |
|  |  |  |  |  |  |  |

*Abbreviations*: ALCOHOL=Daily alcohol intake, g/d; DRUGS=Drug Use; EDUC=Educational attainment (y); HEI=1995 version of the healthy eating index; INSURED=Insured; MAR=Mean Adequacy Ratio; NHANES=National Health and Nutrition Examination Surveys; ns=non-significant; PA=Physical Activity; PIR=Poverty Income Ratio; SMOKE=Smoking behavior; SS=Social Support. See methods for full description of items.

***P<0.001 ; **P<0.01; *P<0.05; ~P<0.10

^1^ Note that sample sizes reflect the person-period data structure rather than participant-level data structure. This analysis was done on the original un-imputed data.

^2^ This path coefficient can be interpreted as Log_e_ of the hazard ratio with time entered as discrete dummy variables for each year of follow-up.

**Table S2.** Total and direct effects of race on all-cause mortality and effects mediated through health-related factors, NHANES III

|  |  | NHB vs. NHW |  | Mexican-American vs. NHW |  |  |
| --- | --- | --- | --- | --- | --- | --- |
| **M=COMORBID** |  | (N=141,488)^1^ |  | (N=142,008)^1^ |  |  |
| Direct effect of race: Race 🡪 Hazard rate^2^ |  | **+0.359±0.052***** |  | +0.051±0.037 |  |  |
| Race 🡪 COMORBID |  | **-0.135±0.006***** |  | **-0.096±0.003***** |  |  |
| COMORBID 🡪 Hazard rate^1^ |  | **+0.187±0.019***** |  | **+0.188±0.020***** |  |  |
| Indirect effect: Race 🡪 COMORBID 🡪Hazard rate |  | **-0.025±0.003***** |  | **-0.018±0.002***** |  |  |
| Mediation proportion, % |  | **-7.60±1.43***** |  | -55.4±62.7 |  |  |
| **M=ALLOSTATIC** |  | (N=122,902)^1^ |  | (N=125,758)^1^ |  |  |
| Direct effect of race: Race 🡪 Hazard rate^2^ |  | **+0.163±0.059**** |  | -0.011±0.040 |  |  |
| Race 🡪 ALLOSTATIC |  | **+0.339±0.001***** |  | **+0.154±0.005***** |  |  |
| ALLOSTATIC 🡪 Hazard rate^1^ |  | **+0.197±0.012***** |  | **+0.202±0.021***** |  |  |
| Indirect effect: Race 🡪 ALLOSTATIC 🡪Hazard rate |  | **+0.066±0.007***** |  | **+0.031±0.004***** |  |  |
| Mediation proportion, % |  | **+29.00±8.00***** |  | >100(ns) |  |  |
| **M=SRH** |  | (N=141,832)^1^ |  | (N=142,442)^1^ |  |  |
| Direct effect of race: Race 🡪 Hazard rate^2^ |  | **+0.159±0.053**** |  | **-0.089±0.038*** |  |  |
| Race 🡪 SRH |  | **+0.272±0.005***** |  | **+0.258±0.003***** |  |  |
| SRH 🡪 Hazard rate^2^ |  | **+0.467±0.032***** |  | **+0.475±0.035***** |  |  |
| Indirect effect: Race 🡪 SRH 🡪Hazard rate |  | **+0.127±0.009***** |  | **+0.123±0.009***** |  |  |
| Mediation proportion, % |  | **+44.40±8.91***** |  | >100(ns) |  |  |

*Abbreviations*: ALLOSTATIC=Allostatic Load; COMORBID=Co-morbid conditions; NHANES=National Health and Nutrition Examination Surveys; ns=non-significant; SRH=Self-Rated Health.

***P<0.001 ; **P<0.01; *P<0.05

^1^ Note that sample sizes reflect the person-period data structure rather than participant-level data structure.

^2^ This path coefficient can be interpreted as Log_e_ of the hazard ratio with time entered as discrete dummy variables for each year of follow-up.
